# Factors associated with success of conservative therapy in chronic subdural hematoma: a single-center retrospective analysis

**DOI:** 10.1007/s00415-024-12307-2

**Published:** 2024-03-30

**Authors:** Merijn Foppen, Roger Lodewijkx, Harssh Verdan Bandral, Kevin Yah, K. Mariam Slot, William Vandertop, Dagmar Verbaan

**Affiliations:** 1 Department of Neurosurgery, Amsterdam Neuroscience, Amsterdam UMC, University of Amsterdam, Room H2-241, Meibergdreef 9, 1105 AZ Amsterdam, The Netherlands; 2 Amsterdam Neuroscience, Neurovascular Disorders, Amsterdam, The Netherlands

**Keywords:** Retrospective studies, Hematoma, Subdural, Chronic, Conservative treatment, Cohort studies, Humans

## Abstract

**Introduction:**

Conservative therapy is a viable option for patients with chronic subdural hematoma (cSDH) who express no, or only mild symptoms. It is not clear which factors are associated with success of conservative therapy. This study aims to determine conservative therapy's success rate and to identify features possibly associated with success.

**Methods:**

A monocenter retrospective cohort study, including cSDH patients treated conservatively (wait-and-watch) from 2012 to 2022, was performed. The primary outcome was success of conservative therapy, defined as ‘no crossover to surgery’ during the follow-up period. Secondary outcomes were (1) factors associated with success, analyzed with univariate and multivariable logistic regression analyses, (2) 30-day mortality (3) time to crossover and (4) reasons for crossover.

**Results:**

We included 159 patients. Conservative therapy was successful in 96 (60%) patients. Hematoma volume (OR 0.79, 95% CI 0.69–0.92) and hypodense hematoma type (OR 3.57, 95% 1.38–9.23) were associated with success. Thirty-day mortality rate was 5% and the median duration between diagnosis and surgery was 19 days (IQR 8–39). Clinical deterioration was the most frequent reason for crossover (in 61/63 patients, 97%) and was accompanied by radiological hematoma progression in 42 patients (67%).

**Conclusion:**

In this selected group of patients, conservative therapy was successful in 60%. Smaller hematoma volume and hypodense hematoma type were associated with success. As time until crossover was approximately three weeks, deploying conservative therapy as primary treatment seems safe and could be rewarding as surgical complications can be avoided. Improvement in patient selection in future cohorts remains warranted.

## Introduction

Chronic subdural hematoma (cSDH) predominantly affects elderly and has an estimated incidence of 8.1–58 per 100,000 per year for patients older than 65 years [1, 2]. Because of the aging population the prevalence of cSDH is expected to increase rapidly in the near future [3]. Clinical presentation can differ concerning the type and severity of symptoms. Some patients experience none, or very mild symptoms.

Surgical therapy is the mainstay of treatment for patients with severe symptoms, such as diminished level of consciousness, hemiparesis or intractable headache [4]. However, especially in this often frail population, surgery comes with complications and an increased risk of mortality and disability, leaving patients dependent on care [5, 6]. Patients with no or relatively mild symptoms, can be managed conservatively by employing a ‘wait-and-watch’ or ‘wait-and-scan’ policy with regular outpatient clinic visits and additional CT-scans if necessary [7]. Strict criteria for conservative or surgical treatment, or general guidelines for cSDH treatment, are not available, leading to considerable treatment variation between hospitals and physicians [8–10].

Existing cSDH literature is mainly focused on surgically treated patients. Studies regarding the effect of conservative therapy are scarce and mostly case reports or series concerning selective, non-consecutive populations [11–31]. A recent systematic review however, reported that success of conservative therapy could be achieved in 60% of all cases [32]. Unfortunately, consistent factors influencing success could not be established. In absence of these parameters, clinicians face challenges in adequately assessing whether the potential benefits and risks of surgery outweigh those of initiating or continuing conservative therapy. This study aims to evaluate the success of initial conservative therapy on a patient level, as well as on hematoma level, as patients with unilateral or bilateral hematoma are not equal, and to identify factors associated with success, in a large consecutive cohort of patients.

## Methods

We performed a single-center, retrospective cohort study at the Amsterdam University Medical Centers, location AMC, a tertiary academic hospital. A list of all consecutive cSDH patients treated at our institution between 2012 and 2022 was comprised. Patients were screened for study eligibility (by MF and RL), using pre-specified in- and exclusion criteria. Patients, aged over 18 years, were included if they had a diagnosis of cSDH on radiological imaging, confirmed by a neuroradiologist, and if the primary treatment was conservative therapy, defined as ‘wait-and-watch therapy’. If there was uncertainty about whether a patient was eligible for inclusion, a third adjudicator (MS, neurosurgeon) was consulted for the final decision. Patients were excluded if conservative treatment contained medication (steroids or tranexamic acid) or any intervention (middle meningeal artery embolization), if they were included in an ongoing randomized controlled trial (TORCH-study [33]), if the cSDH developed after a craniotomy, if a cerebrospinal fluid shunt was in situ, or in case of withdrawal of therapy. In general, anticoagulant (AC) and antiplatelet (AP) therapy was discontinued at diagnosis. Only if there was a strong indication, such as a mechanical heart valve or recent myocardial or cerebral infarction, AC and AP therapy were continued. The local ethics committee determined that this study did not fall under the Medical Research Involving Human Subjects Act (WMO), and we obtained a waiver for official ethical approval (waiver number: W22_098#22.136).

### Outcomes

The primary outcome was success of conservative therapy, defined as ‘no crossover to surgery’ during the follow-up period. Conservative therapy was also deemed unsuccessful for patients with a bilateral cSDH if unilateral surgical evacuation was performed during follow-up. In order to study hematoma-specific characteristics, success of conservative therapy was also analyzed per hematoma. For this analysis, patients with a bilateral hematoma were considered as two separate cases and success of conservative therapy could be achieved per hematoma. Secondary outcomes were factors associated with success of conservative therapy, 30-day mortality rate and for those who crossed over to surgery, the time-to-crossover and reasons for crossover to surgery.

### Data collection

**Fig. 1:**
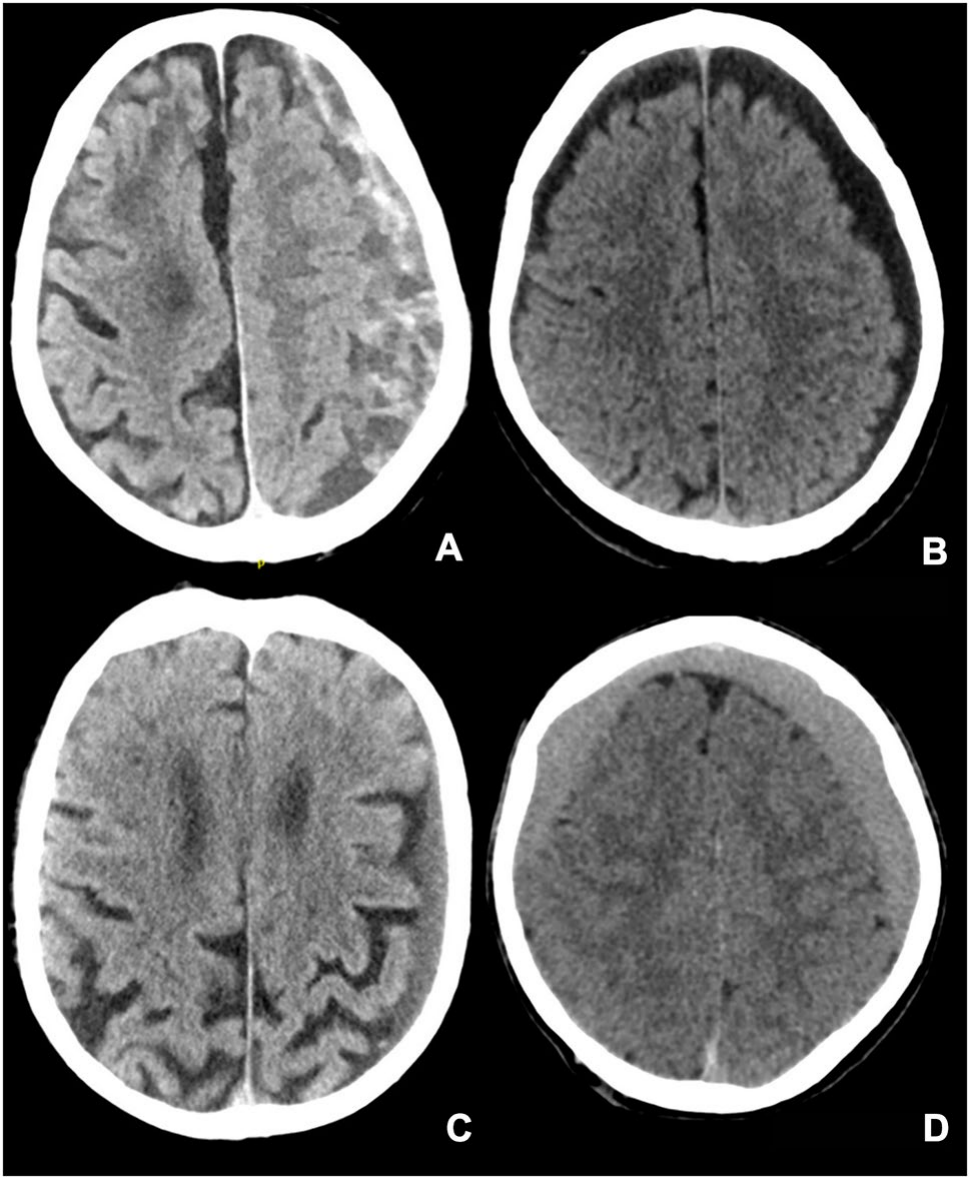
Classification of hematoma type. **A** mixed type hematoma, **B** homogeneous hypodense hematoma, **C** homgeneous isodense hematoma, **D** homogeneous hyperdense hematoma

**Fig. 2:**
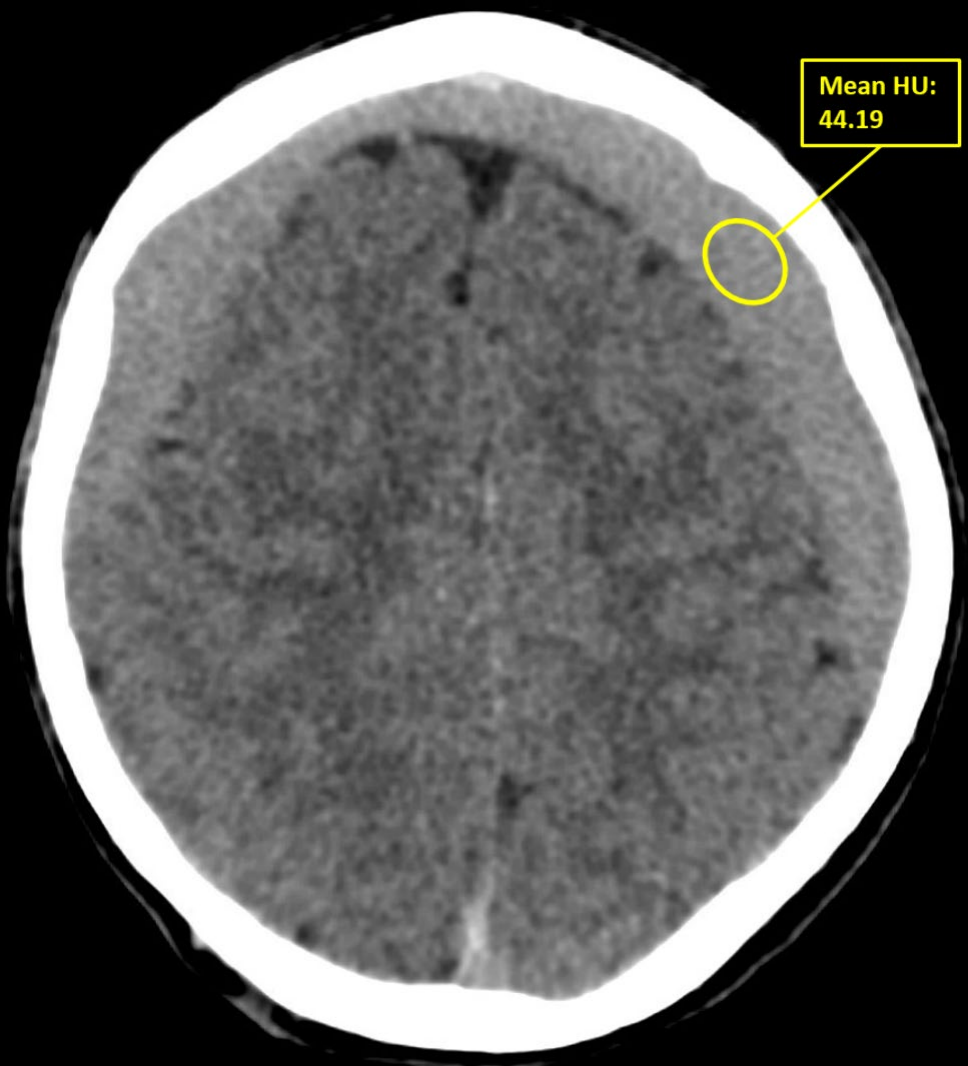
Method of assessing average amount of HU for homogeneous hematomas

From the patients’ medical file we retrieved the patients demographics, medical history, use of anticoagulant or antiplatelet therapy, statins or ACE-inhibitors and clinical features at diagnosis, as well as reasons for crossover to surgery, the time between diagnosis and eventual surgery and 30-day mortality. An aSDH in history was defined as ‘an aSDH in the year prior to diagnosis of cSDH located on the same convexity as the cSDH’. Patients were classified as having a motor deficit at diagnosis if they exhibited any form of motor function loss (according to the Medical Research Council scale (MRC)). Radiological characteristics retrieved from reports included hematoma laterality, maximum diameter and presence and amount of midline shift. If one or multiple of these radiological variables were not described in the radiological report, these parameters were measured manually, as was hematoma volume using Brainlab software (Brainlab AG, Munchen, Germany) and hematoma type. Hematomas were categorized as either mixed or homogeneous [31, 34]. The homogeneous type was further classified based on Hounsfield Units (HU) into hypodense (HU < 25), isodense (HU 25–35) and hyperdense (HU > 35) hematomas (see Figs. 1 and 2). Data were stored using an online database (Castor EDC).

After diagnosis, patients were followed either by a neurologist or a neurosurgeon. Follow-up was primarily performed in our hospital, but in a small fraction of patients, follow-up was performed by the referring neurologist. Duration of follow-up was calculated from the date of diagnosis until date of last available outpatient clinic visit, or date of the last available head CT-scan. If follow-up data from another hospital was available, this was used.

### Statistical analysis

Patient characteristics were compared using parametric and non-parametric tests. The normality of continuous variables was assessed with the Shapiro–Wilk test and considered normally distributed with a value > 0.9. A mean standard deviation (SD) were calculated for normally distributed variables. A median and interquartile range (IQR) was calculated for not normally distributed variables. Baseline characteristics between both groups (success vs. crossover) were compared using appropriate tests (Mann–Whitney *U* test, Chi-squared test, Fisher’s exact test and independent samples *t* test). Missing baseline values (see Table 1) were imputed 20 times using chained equations, assuming a missing at random (MAR) pattern. All available baseline variables, three auxiliary variables (center and year of diagnosis and presence of an arachnoid cyst) and the outcomes success of conservative therapy and 30-day mortality were used to impute missing values. Predictors for the success of conservative treatment per patient were assessed with univariate and multivariable logistic regression models. Variables with p < 0.2 in univariate analyses were included in the multivariable regression model. The pooled estimates across 20 imputed datasets of the multivariable analyses were calculated using Meng and Rubin’s rules. A similar, separate analysis, was performed to determine success of conservative therapy per hematoma. For this analysis, hematoma laterality (uni- vs. bilateral hematoma), hematoma type, hematoma diameter and volume were used to impute missing hematoma baseline values. All statistical analyses were performed with IBM SPSS statistics, version 28.0 and R version 4.2.1 (R Foundation for Statistical Computing, Vienna, Austria; http://www.R-project.org/) [35–37].

**Table 1:** Baseline characteristics of 159 cSDH patients treated with conservative therapy

Variable	Total (n = 159)	Success (n = 96)	Crossover (n = 63)	p-values
Age (sd)	70.5 (12.8)	69.6 (13.3)	71.8 (12.4)	0.280^a^
Male (%)	117 (73.5)	71 (73.9)	46 (73.0)	0.895^b^
*History*				
Arrhythmia (%)^158^	29 (18.3)	16 (16.8)	13 (20.6)	0.547^b^
Cerebrovascular accident (%)^158^	33 (20.8)	21 (22.1)	12 (19.0)	0.643^b^
Ischemic heart disease (%)^158^	24 (20.8)	16 (16.8)	8 (12.7)	0.477^b^
DVT or PE (%)^158^	11 (7.5)	5 (5.3)	6 (9.5)	0.348^d^
COPD (%)^158^	12 (7.5)	9 (9.5)	3 (4.8)	0.365^d^
Diabetes (%)^158^	39 (24.7)	26 (27.3)	13 (20.6)	0.337^b^
Hypertension (%)^158^	67 (42.4)	46 (48.4)	21 (33.3)	0.060^b^
Malignancy (%)^158^	35 (22.2)	24 (25.3)	11 (17.5)	0.248^b^
Alcoholism in history (%)^158^	15 (9.5)	10 (10.5)	5 (7.9)	0.587^b^
Head trauma (%)^155^	105 (67.7)	64 (67.4)	41 (68.3)	0.900^b^
aSDH prior to cSDH (%)	18 (11.3)	14 (14.6)	4 (6.3)	0.109^b^
*Medication*				
AC or AP (%)^158^	70 (44.3)	41 (43.2)	29 (46.0)	0.722^b^
Anticoagulation therapy	28	15	13	
Antiplatelet therapy	41	25	16	
Both AC and AP	1	1	0	
Statins	60 (38.0)	37 (38.9)	23 (36.5)	0.887^b^
ACE-inhibitors	35 (22.2)	22 (23.2)	13 (20.6)	0.859^b^
*Clinical features at diagnosis*				
GCS (IQR) ^144^	15 (14.8–15)	15 (14–15)	15 (15–15)	0.216^e^
Headache (%)^149^	71 (47.7)	38 (42.7)	33 (55.0)	0.140^b^
Motor deficit (%)^145^	19 (11.9)	11 (11.5)	8 (12.7)	0.862^b^
Pronation, drift or fall of arm/leg	6 (31.6)	3 (27.3)	3 (37.5)	
MRC 4	12 (63.2)	7 (63.6)	5 (62.5)	
MRC 2	1 (5.3)	1 (9.1)	0 (0.0)	
Markwalder grading scale^151^				0.432^d^
0	35 (23.2)	25 (27.2)	10 (16.9)	
1	74 (49.0)	41 (44.6)	33 (55.9)	
2	40 (26.5)	24 (26.1)	16 (27.1)	
3	1 (0.7)	1 (1.1)	0 (0.0)	
4	1 (0.7)	1 (1.1)	0 (0.0)	
*Radiological characteristics*				
Unilateral cSDH’s (%)	95 (59.7)	63 (65.6)	32 (50.8)	0.062^b^*
Left-sided cSDHs (%)	44 (27.7)	27 (28.1)	17 (27.0)	
Right-sided cSDHs (%)	51 (32.1)	36 (37.5)	15 (23.8)	
Bilateral cSDHs (%)	64 (40.3)	33 (34.4)	31 (49.2)	
Midline shift (%)^158^	92 (58.2)	53 (55.2)	39 (62.9)	0.338^b^
Midline shift in mm (median)^157^	3 mm (0–5)	2 mm (0–5)	3 mm (0–6)	0.232^b^
Hematoma diameter in mm (mean)^155*¥*^	13.7 mm (6.9)	13.0 mm (6.9)	14.8 mm (7.0)	0.110^a^
Hematoma volume in cl (mean)^150*¥*^	7.1 (+ − 3.7)	6.2 (+ − 3.5)	8.5 ml (+ − 3.7)	< 0.001^a^

With superscript in the column, ‘variable’ is indicated for how many patients data was available if data of one or more patient(s) was missing

*Unilateral vs. bilateral hematoma, ^a^Independent *t* test, ^b^Chi-squared test, ^d^Fishers-exact test, ^e^Mann-Whitney *U* test

^¥^In patients with a bilateral cSDH, the hematoma with the maximum diameter or volume was used for analysis. Anticoagulant and antiplatelet therapy was ceased in 64 (91.4%) patients

## Results

### Baseline characteristics

A total of 159 patients were included. The mean age of all patients was 70.5 years (SD 12.8). Of these 117 (74%) were male (Table 1). Nineteen (11.9%) patients had motor deficits at diagnosis. All patients, except one, had only a mild form (MRC > 3) of motor deficit. The patient with severe muscle weakness most likely suffered Todd’s paralysis since it appeared after a seizure. The weakness improved gradually after treatment with antiepileptic medication. Two patients had a MGS score of more than two at diagnosis. This high grade was based on multi-morbidity as one patient presented with additional pneumothorax, costal fractures and cerebral contusion, and one patient had concomitant skull fractures with cerebral contusions, costal fractures and a scapula fracture. Ninety-five (60%) patients had a unilateral cSDH and 64 patients had a bilateral hematoma. Thus, a total of 223 hematomas were included. Anticoagulant or antiplatelet therapy was discontinued in 64 out of 70 patients. The characteristics per hematoma are described in Table 2. Midline shift was present in 92 (58%) cases, with a median of 3 mm (IQR 0–5). Notably, nine of these cases had a shift exceeding 10 mm. Mean hematoma diameter and mean hematoma volume were 13.7 mm (SD 6.9) and 7.1 cl (SD 3.7), respectively.

**Table 2:** Univariate analysis per hematoma

Variable	Total hematomas (n = 223)	Success (n = 144)	Crossover (n = 79)	p-values
Unilateral cSDH (%)	95 (42.7)	63 (43.8)	32 (40.5)	0.639^b^
*Type*^*186*^				0.005^b^
Mixed (%)	78 (41.9)	41 (35.8)	37 (54.4)	
Homogeneous (%)				
Hypodense(%)	49 (26.3)	40 (33.3)	9 (13.2)	
Isodense (%)	28 (15.1)	20 (16.7)	8 (11.8)	
Hyperdense (%)	31 (16.7)	17 (14.2)	14 (20.6)	
Hematoma diameter in mm (sd)^218^	12.5 (6.8)	11.4 (6.5)	14.5 (6.7)	0.001^a^
Hematoma volume in cl (sd)^208^	6.5 (3.7)	5.5 (3.4)	8.3 (3.6)	<0.001^a^

With superscript in the column, ‘variable’ is indicated for how many patients data was available if data of one or more patient(s) was missing

^a^Independent *t* test, ^b^Chi-squared test

### Success rate and factors associated with success

Success of conservative therapy was achieved in 96 (60%) patients. Patients in the success group had a significantly smaller hematoma volume than in the crossover group (6.2 centiliters vs. 8.5 centiliters, p < 0.001), see Table 1. In multivariable regression analysis, only smaller hematoma volume was associated with success of conservative therapy (OR 0.79 95% CI 0.69–0.92, Table 3). Success of conservative therapy was achieved in 142 hematomas (64%). In multivariable regression analysis, hypodense hematoma type (OR 3.57, 95% CI 1.38–9.23) and volume (OR 0.76, 95% CI 0.67–0.87) were associated with success of conservative therapy (Tables 2 and 3).

**Table 3:** Multivariable analysis of variables associated with success

Variable	OR	95% CI
*Analysis per patient*		
Hypertension	1.832	0.865–3.881
aSDH	3.093	0.807–11.861
Headache	1.661	0.808–3.418
Unilateral hematoma	0.766	0.365–1.605
Hematoma diameter	1.057	0.983–1.137
Hematoma volume (per cl)	0.795	0.689–0.917*
*Analysis per hematoma*		
Mixed type		Reference category
Hypodense type	3.574	1.383–9.238
Isodense type	1.732	0.645–4.653
Hyperdense type	0.898	0.338–2.383
Hematoma diameter	1.047	0.981–1.119
Hematoma volume (per cl)	0.760	0.678–0.872*

*Statistically significant

### Other outcomes

The median follow-up in the total study population was 74 days (IQR 28–133). The median follow-up of the success group was shorter than the crossover group (52 vs. 79 days). Seven (5%) patients died within 30 days after diagnosis (7.6% in the success group vs. 1.6% in the crossover group, p = 0.135). Five patients died from a cause not directly related to the cSDH (two due to sepsis, one aspiration pneumonia, one respiratory insufficiency and one gastrointestinal bleeding due to a peptic ulcer with aspiration). Four of these were initially hospitalized because of their cSDH. In two patients, the cause of death remained unknown. In the crossover group, clinical deterioration (61/63 (97%) patients) was the most common reason for surgery (Table 4). In those patients, clinical deterioration was accompanied by radiological progression of the hematoma in 42 patients (67%). Notably, pre-operative imaging was not repeated for all patients (n = 5, 8%) with clinical deterioration. For these patients, the average period between diagnosis and surgery was five days. In two patients, radiological growth of the hematoma was the only reason for crossover to surgery. In one of these patients an additional argument for surgery was that the neurological symptoms at diagnosis did not resolve over time. The median period between diagnosis and surgery was 19 days (IQR 8–39).

**Table 4:** Reasons for crossover to surgery

Reason for crossover	Number of patients (%)
Multiple symptoms	31 (49.2)
GCS	8 (12.7)
Language disorder	6 (9.5)
Gait disorder	6 (9.5)
Headache	5 (7.9)
Motor deficit	2 (3.2)
Papilledema	1 (1.6)
Epileptic seizures	1 (1.6)
Cognitive complaints	1 (1.6)
Radiological progression	2 (3.2)

A decrease in Glasgow Coma Scale (GCS) led to surgery in eight patients. For all other symptoms, an increase in severity resulted in surgery. For 31 patients this was a combination of multiple symptoms. In two patients, radiological progression only was the reason for crossover

## Discussion

In this retrospective, single-center cohort study, conservative treatment was successful in 60% of selected cSDH patients with no, or only mild, symptoms. Small hematoma volume and hypodense hematoma type were associated with success of conservative therapy.

Crossover rates reported in the literature are based on case reports, case series, and small retrospective cohort studies, but large prospective series are missing. Consequently, crossover rates vary considerably, from 0–7% [23, 25] to 84–93% [16, 22]. The main reason for this variation is selection bias, leading to study population heterogeneity. Some studies exclude patients with large hematomas or those with significant mass effect, resulting in a low crossover rate [25]. Other studies include only patients with a midline shift greater than 10 mm, resulting in a high crossover rate [22]. Severity of neurological deficits (quantified by MGS or GCS) is another critical factor influencing the crossover rate. In studies reporting crossover rates for patients with a maximum GCS or minimal MGS, rates are as low as 0–1%) compared to studies that include more severely affected patients [23, 30]. These variable in- and exclusion criteria complicate direct comparisons and prohibit drawing definitive conclusions. Our study did not apply these specific criteria as we aimed to quantify the crossover rate in a population representative of a significant portion of cSDH patients. Therefore, we argue that the crossover rate found in this study is a more accurate estimation than reported in existing studies.

Hematoma volume has been reported as a potential factor in predicting success of conservative therapy. The results, however, are conflicting. In a 2016 study by Kim et al., the group successfully treated with conservative therapy had a smaller hematoma volume, though the difference was not statistically significant (p = 0.146) [19]. In 2022, Wang et al. published one of the few larger studies focusing on predictive parameters for the outcome of conservative therapy [24, 27]. Among 98 patients, larger hematoma diameter and volume were significantly associated with crossover to surgery (thickness: OR 1.097, volume: OR 1.021). However, these results stem from a highly pre-selected population given that the data originated from a RCT. The study excluded patients on anticoagulant or antiplatelet medication, representing up to 50% of all cSDH patients [38–40]). Those on statins, accounting for approximately 30% of all cSDH patients, were also omitted. [40, 41]). Nonetheless, our study corroborates that hematoma volume influences the success of conservative therapy, even in a broader population.

Hypodense hematoma type was another factor associated with conservative therapy success. The underlying theory is that in hypodense hematomas, active bleeding components are absent [42, 43]. Therefore, hematoma growth and eventual surgery are not likely. Two studies have reported a positive association between hypodense hematoma density and success of conservative therapy [16, 31]. Unfortunately, the first study published did not measure HU, leading to methodological subjectivity and limited reproducibility [16]. In the second study, the method of hematoma classification was identical to the one used in this study [31]. A recent systematic review indicated that the major benefit of this classification method is its simplicity, especially in comparison to other, more detailed, available methods [44]. This simplicity reduces interobserver variability and enhances data consistency across studies. Given its straightforward implementation and precedent in prior research, we recommend that future studies adopt a similar classification approach.

Our study also shows that progression to surgery was a subacute process and did not occur directly or in the days shortly after diagnosis of the cSDH. The time until crossover found in this study concurs with a study by Jiang et al. and Rauhala et al. [24, 45]. The first study was a RCT in which the effect of atorvastatin on the crossover to surgery rate was investigated. In the placebo group, the mean time until crossover to surgery was 24 days (n = 98) [24]. In the second study, 223 patients were initially treated with conservative therapy, of whom 53 (24%) eventually required surgery. The average time between diagnosis and surgery was 24 days [45]. Considering the aforementioned study results and the provided time frame of this study, we conclude that wait-and-watch therapy can be safely deployed as initial treatment for this group of cSDH patients.

While neurologists and neurosurgeons at our institution collaborate closely for cSDH treatment, the decision for surgery lies with the attending neurosurgeon. Studies have shown that this decision is predominantly based on subjective features such as treatment center culture, surgeon preference and intuition [46, 47]. Clinical deterioration, frequently paired with radiological progression, was the prevalent reason for crossover in our study. Interestingly, motor deficit at diagnosis, which most surgeons often interpret as an indication for surgery, was evenly distributed amongst both groups. This implies that presence of (mild) motor deficits at diagnosis is not an indication for surgery alone. However, the total group of patients in which motor deficit was present was relatively small, so caution is required with interpretation of these results. Secondly, nine patients had a midline shift exceeding 10 mm at diagnosis. Five of these required eventual surgery, but in four success of conservative therapy was achieved. Deciding optimal treatment strategy is complex, especially in mildly symptomatic patients with such a midline shift. In order to prevent potential rapid neurological decline due to further brain herniation, most neurosurgeons would perform surgery. However, even in these cases success of conservative therapy can be achieved. Again, with such limited number of patients vast conclusions cannot be drawn and future research specifying indications for surgery is warranted.

### Limitations

The most important limitation of this study is lack of clinical outcome and other outcomes related to functioning, independence or quality of life. Since this is a retrospective study, reliable assessment of such outcomes (e.g. through modified Rankin scale, mNIHSS, SF-36, or Barthel index) was not adequately documented. Secondly, because this study was conducted in a tertiary academic hospital, the results cannot be extrapolated directly onto general cSDH populations. Asymptomatic patients might have been overlooked, as they typically get managed by neurologists in referring centers instead of being sent to our institution. Therefore, the success rate found in this study probably underestimates the true success rate. Finally, the success group had a shorter follow-up compared to the crossover group. This reflects current standard care as patients were either referred back to the neurologist, or discharged of follow-up with instructions to regain contact if symptoms re-occurred or worsened. Standardized follow-up could solve these issues in future prospective and multi-centered studies.

## Conclusion

In this single-center, retrospective study, conservative therapy of patients with a cSDH and no, or only mild, symptoms could be achieved without crossover-to-surgery in a slight majority of patients, even in the presence of (mild) motor deficits. Parameters associated with success were hematoma volume and hypodense hematoma type. As time to crossover in these selected patients is in the order of weeks, cautious follow-up appears to be safe and feasible. In order to determine the true efficacy of conservative therapy, especially considering clinical outcomes, future prospective studies are necessary. This requires a standardized, nationwide (and preferably international), and integrated approach.

## Data Availability

Not applicable.

## Abbreviations

AC: Anticoagulant therapy
AP: Antiplatelet therapy
aSDH: Acute subdural hematoma
BHC: Burr hole craniostomy
CI: Confidence interval
cSDH: Chronic subdural hematoma
COPD: Chronic obstructive pulmonary disease
CVA: Cerebrovascular accident
CT: Computed tomography
CL: Centiliter
eCRF: Electronic case report form
DVT: Deep venous thrombosis
EDC: Electronic data capture
HU: Hounsfield Units
IQR: Interquartile range
GCS: Glasgow coma scale
MAR: Missing at random
MGS: Markwalder Grading scale
MRC: Medical Research Council scale
MM: Millimeter
ML: Milliliter
OR: Odds ratio
PE: Pulmonary embolism
RCT: Randomized controlled trial
SD: Standard deviation
PE: Pulmonary embolism
SD: Standard deviation
